# Description of a new species of *Paraplehnia* (Polycladida, Stylochoidea) from Japan, with inference on the phylogenetic position of Plehniidae

**DOI:** 10.3897/zookeys.864.33955

**Published:** 2019-07-15

**Authors:** Yuki Oya, Taeko Kimura, Hiroshi Kajihara

**Affiliations:** 1 Graduate School of Science, Hokkaido University, Sapporo 060-0810, Japan; 2 Graduate School of Bioresources, Mie University, Kurimamachiya-cho 1577, Tsu 514-8507, Japan; 3 Faculty of Science, Hokkaido University, Sapporo 060-0810, Japan

**Keywords:** 28S rDNA, Acotylea, Bayesian inference, COI, marine flatworm, maximum likelihood, taxonomy

## Abstract

We describe a new species of polyclad flatworm, *Paraplehniaseisuiae***sp. nov.**, from 298–310 m depths in the Sea of Kumano, West Pacific, Japan. *Paraplehniaseisuiae***sp. nov.** is characterized by i) a developed muscular wall proximally occupying about one-third of the prostatic vesicle, ii) no common duct between the spermiducal bulbs and the prostatic vesicle, and iii) a genital pit between the male and female gonopores. We provide a partial sequence (712 bp) of the mitochondrial cytochrome *c* oxidase subunit I gene as a DNA barcode for the species. Our phylogenetic analyses based on 603-bp 28S rDNA sequences indicate that *P.seisuiae***sp. nov.** is nested in a clade consisting of stylochoid species along with unidentified species of *Stylochus*. It suggests that Plehniidae belongs to Stylochoidea, although this should be confirmed by future studies that contain *Plehniaarctica* (Plehn, 1896), the type species of the type genus of the family. The interfamily relationship among the superfamily Stylochoidea remains poorly resolved.

## Introduction

Polyclad flatworms in the family Plehniidae Bock, 1913 are characterized by possessing sperm ducts or a common sperm duct entering the neck of a prostatic vesicle, the latter lacks an ejaculatory duct (Hyman 1953). The majority of plehniids have been reported from sublittoral zones by dredging (e.g. Bock 1913, 1923; Kato 1939; Hyman 1953; Hagiya 1993); some species were described from more than 200 m depths (Bock 1913; Hyman 1953).

The superfamilial affinity of Plehniidae has not been molecularly tested, while both Faubel (1983) and Prudhoe (1985) placed the family in Stylochoidea Poche, 1926 based on morphological characters. No plehniid has been represented in recent molecular phylogenetic analyses (Aguado et al. 2017; Bahia et al. 2017; Tsunashima et al. 2017; Litvaitis et al. 2019), although Bahia et al. (2017) indicated that Plehniidae possibly belongs to Cryptoceloidea Laidlaw, 1903. There has been a conflict between Faubel (1983) and Prudhoe (1985) as to the genus-level classification in Plehniidae. Faubel (1983) divided Plehniidae into three genera: *Diplehnia* Faubel, 1983, *Discocelides* Bergendal, 1893, and *Plehnia* Bock, 1913. Prudhoe (1985) separated this family into four genera: *Discocelides*, *Nephtheaplana* Prudhoe, 1985, *Paraplehnia* Hyman, 1953, and *Plehnia*. Later, Newman and Cannon (1997) established a new genus, *Myoramyxa*, within Plehniidae in the sense of Prudhoe (1985).

In this paper, we describe a new species of plehniid flatworm from Japan. We provide a partial sequence of the cytochrome *c* oxidase subunit I (COI) gene as a DNA barcode for the new species. We estimate the phylogenetic position of Plehniidae, represented by the new species, among other acotylean polyclads by molecular analyses using partial 28S rDNA sequences.

## Material and methods

A single polyclad specimen was collected by dredging during the research cruise No. 1722 by Training/Research Vessel (TRV) *Seisui-maru*. The worm was anesthetized in a MgCl_2_ solution prepared with tap water so that it had the same refractive index (or “salinity”) as the seawater, using an IS/Mill-E refractometer (AS ONE, Japan), and then photographed with a Nikon D5300 digital camera with external strobe lighting provided by a pair of Morris Hikaru Komachi Di flash units. For DNA extraction, a piece of the body margin was cut away from the specimen and fixed in 100% ethanol. The rest of the body was fixed in Bouin’s solution for 24 h and preserved in 70% ethanol. It was then cut into two (anterior and posterior) pieces. Both pieces were dehydrated in an ethanol series and cleared in xylene, then embedded in paraffin wax, sectioned at 7 µm thickness, stained with hematoxylin and eosin (HE), and embedded in Entellan New (Merck, Germany). They were observed under an Olympus BX51 compound microscope and photographed with a Nikon D5300 digital camera.

Sections containing part of copulatory apparatus, mounted on one of the slides, were re-stained by Mallory’s trichrome method to yield clear contrast between the muscular and connective tissues. The cover glass was removed by steeping the preparation in xylene for 24 h. The sections on the slide were hydrated in an ethanol series. HE staining was then removed by washing in 50% ethanol containing 0.5% HCl for 2 h. After Mallory’s staining, the sections were likewise embedded in Entellan New.

Total DNA was extracted by using Boom et al.’s (1990) silica method. A fragment of the cytochrome *c* oxidase subunit I (COI) (712 bp) was amplified with primers Acotylea_COI_F and Acotylea_COI_R (Oya and Kajihara 2017) as a reference for DNA barcoding. A 1004-bp fragment of 28S rDNA was amplified with primers fw1 and rev2 (Sonnenberg et al. 2007) for molecular phylogenetic analyses; the primer pairs have been used in other phylogenetic studies of polyclads (e.g. Bahia et al. 2017; Litvaitis et al. 2019). Polymerase chain reaction (PCR) amplification conditions were 94 °C for 5 min; 35 cycles of 94 °C for 30 s, 50 °C (COI) or 52.5 °C (28S rDNA) for 30 s, and 72 °C for 1.5 min (COI) or 2 min (28S rDNA); and 72 °C for 7 min. All nucleotide sequences were determined by direct sequencing with a BigDye Terminator Kit ver. 3.1 and 3730 Genetic Analyzer (Life Technologies, California, USA); two internal primers, fw2 and rev4 (Sonnenberg et al. 2007), were used in sequencing 28S rDNA. Sequences were checked and edited using MEGA ver. 5.2 (Tamura et al. 2011).

Additional 28S rDNA sequences of Acotylea were downloaded from GenBank. Two cotylean species were chosen as outgroups (Table 1): *Cestoplanarubrocincta* (Grube, 1840) and *Pericelisorbicularis* (Schmarda, 1859), the former was transferred to Cotylea by Bahia et al. (2017). Sequences were aligned using MAFFT ver. 7 (Katoh and Standley 2013), with the FFT-NS-i strategy selected by the “Auto” option. Ambiguous sites were removed with Gblocks ver. 0.91b (Castresana 2002) using a less stringent option. The optimal substitution models selected with Kakusan4 (Tanabe 2011) under the Akaike Information Criterion (AIC) (Akaike 1974) were GTR+G.

**Table 1.** List of species that were used for the molecular phylogenetic analysis and respective GenBank accession numbers.

Species	GenBank accession number
Acotylea	
*Adenoplanaevelinae* Marcus, 1950	KY263647
*Amemiyaiapacifica* Kato, 1944	LC100077
*Armatoplanaleptalea* (Marcus, 1947)	KY263649
*Callioplanamarginata* (Stimpson, 1857)	LC100082
*Discoplanagigas* (Schmarda, 1859)	LC100080
*Echinoplanacelerrima* Haswell, 1907	HQ659020
*Hoploplanacalifornica* Hyman, 1953	KC869850
*Hoploplanadivae* Marcus, 1950	KY263692
*Hoploplanavillosa* (Lang, 1884)	LC100076
*Idioplanaaustraliensis* Woodworth, 1898	HQ659008
*Imogineijimai* (Yeri & Kaburaki, 1918)	LC100079
*Imogineoculiferus* (Girard, 1853)	HQ659007
*Imoginerefertus* (Du Bois-Reymond Marcus, 1965)	KY263694
*Imoginezebra* (Verrill, 1882)	AF342800
*Koinostylochuselongatus* (Kato, 1937)	LC100083
*Leptoplanatremellaris* (Müller, 1773)	KY263696
*Leptostylochusgracilis* Kato, 1934	LC100078
*Melloplanaferruginea* (Schmarda, 1859)	HQ659014
*Notocomplanahumilis* (Stimpson, 1857)	LC100085
*Notoplanaaustralis* (Schmarda, 1859)	HQ659015
*Notoplana* sp.	KY263651
*Paraplanoceraoligoglena* (Schmarda, 1859)	KC869849
*Paraplanocera* sp.	KY263699
*Paraplehniaseisuiae* sp. nov.	LC467000
*Phaenocelismedvedica* Marcus, 1952	KY263706
*Planoceramultitentaculata* Kato, 1944	LC100081
*Pleioplanadelicata* (Yeri & Kaburaki, 1918)	LC100088
*Pseudostylochusobscurus* (Stimpson, 1857)	LC100084
*Stylochus* sp.	KY263743
Outgroup (Cotylea)	
*Cestoplanarubrocincta* (Grube, 1840)	HQ659009
*Pericelisorbicularis* (Schmarda, 1859)	EU679116

Phylogenetic analyses were performed with maximum-likelihood (ML) methods and Bayesian Inference (BI). The ML analysis was performed with RAxML ver. 8.2.3 (Stamatakis 2014). Nodal support within the ML tree was assessed by analyses of 1,000 bootstrap pseudoreplicates (Felsenstein 1985). BI was performed with MrBayes ver. 3.2.2 (Ronquist and Huelsenbeck 2003). The Markov chain Monte Carlo (MCMC) process used random starting trees and involved four chains for 1,000,000 generations. The first 25% of the trees were discarded as burn-in.

Type slides have been deposited in the Invertebrate Collection of the Hokkaido University Museum, Sapporo, Japan (ICHUM). The sequences determined in this study have been deposited in DDBJ/EMBL/GenBank databases with the accession numbers LC466999 (COI) and LC467000 (28S rDNA).

## Results

### Family Plehniidae Bock, 1913 sensu Prudhoe (1985)

#### Genus *Paraplehnia* Hyman, 1953

##### Paraplehnia seisuiae sp. nov.

Taxon classificationAnimaliaPolycladidaPlehniidae

http://zoobank.org/565559B6-CFC2-4CF6-A4F7-BFAFCCF4EEDA

Figures 1 , 2

###### Etymology.

The specific name is a noun in the genitive case and taken from the TRV*Seisui-maru*.

###### Material examined.

One specimen: holotype, ICHUM 5345, 44 slides (14 slides for the anterior part and 30 slides for the posterior part of the body), dredged from 298–310 m depths, the Sea of Kumano, between 34°08.0'N, 136°37.8'E to 34°07.8'N, 136°37.9'E, Japan.

###### Description.

**Figure 1.**
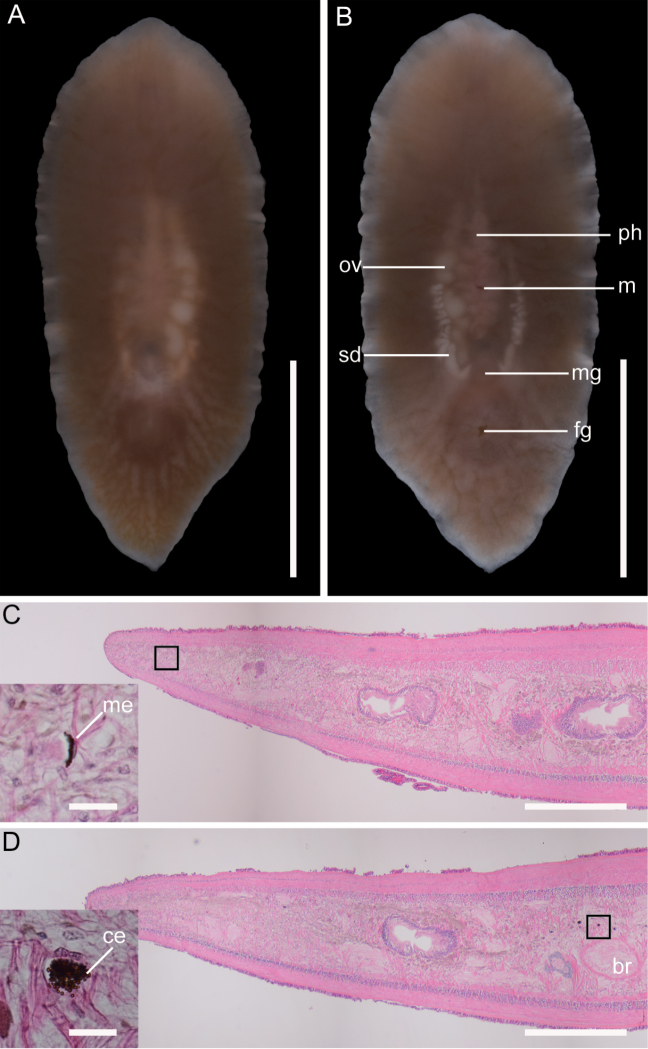
*Paraplehniaseisuiae* sp. nov., ICHUM 5345 (holotype), photographs taken in life and eyespots observed in sections. **A** Dorsal view **B** ventral view **C** marginal eyespot (inset showing magnification of black box) **D** cerebral eyespot (inset showing magnification of black box). Abbreviations: **br** brain **ce** cerebral eyespot **fg** female gonopore **m** mouth **me** marginal eyespot **mg** male gonopore **ov** oviduct **ph** pharynx **sd** sperm duct. Scale bar: 10 mm (**A**, **B**); 600 μm (**C**, **D**); 20 μm (insets in **C**, **D**).,

Live specimen 26 mm in length, 11 mm in width. Body thick, elongate, oval, narrow toward posterior end (Fig. 1A, B). Anterior and posterior ends pointed. Body ground color translucent to whitish opaque. General appearance of body light brown. Dorsal body without any pattern. Body margin translucent. Tentacles lacking. Pharynx, ruffled in shape, 7.4 mm in length, located at center of body. Mouth opening at center of pharyngeal cavity (Fig. 1B). Intestine highly branched and not anastomosing, spreading throughout body except margin. Pair of sperm ducts and oviducts whitish, visible through ventral surface. Male and female gonopores separate; male gonopore opening at 9 mm from posterior end; female gonopore situated 2.5 mm posterior to male gonopore.

Marginal and cerebral eyespots small and embedded in parenchyma (Fig. 1C, D). At least 47 and 28 eyespots arranged in anterior body margin and from just behind brain to anterior to brain, respectively, but detailed distribution of eyespots could not be observed.

**Figure 2.**
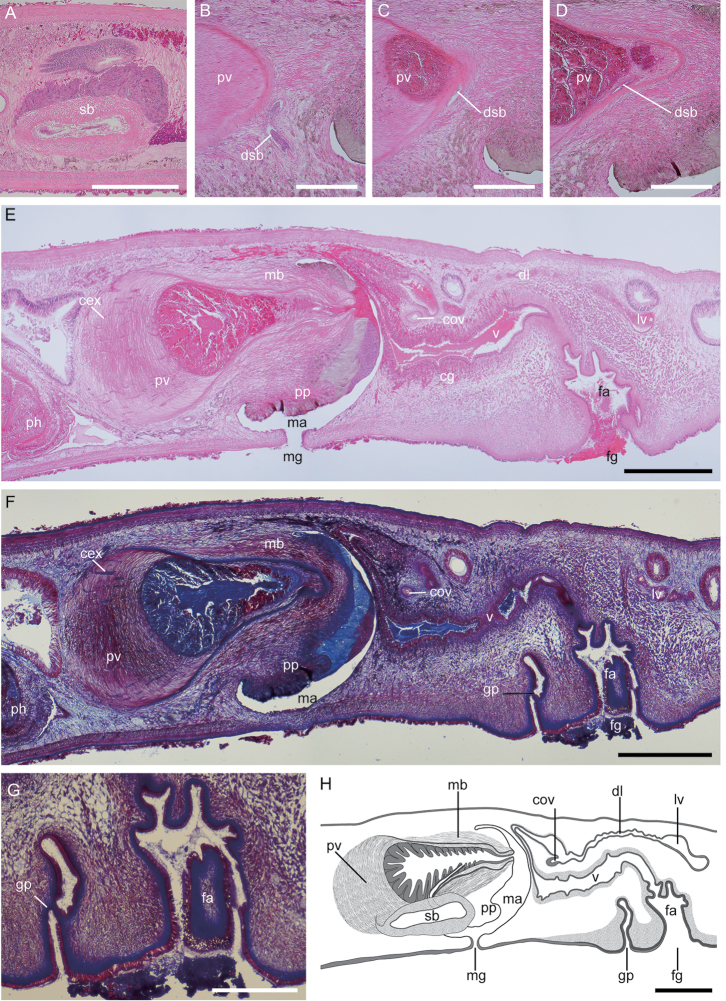
*Paraplehniaseisuiae* sp. nov., ICHUM 5345 (holotype), sagittal sections (**A–G**) and schematic diagram (**H**). **A** Spermiducal bulb **B–D** connection between spermiducal bulb and prostatic vesicle **E**, **F** copulatory apparatus **G** genital pit **H** schematic diagram of copulatory complex. Abbreviations: **cex** canal of extra-vesicular grand **cg** cement glands **cov** common oviduct **dl** duct of Lang’s vesicle **dsb** duct of spermiducal bulb **fa** female atrium **fg** female gonopore **gp** genital pit **lv** Lang’s vesicle **ma** male atrium **mb** muscular bulb **mg** male gonopore **ph** pharynx **pp** penis papilla **pv** prostatic vesicle **sb** spermiducal bulb **v** vagina. Scale bars: 600 μm (**A**, **E**, **F**, **H**); 300 μm (**B–D**, **G**). Staining: hematoxylin and eosin stain (**A–E**); Mallory’s triple stain (**F**, **G**).

Male copulatory apparatus located posteriorly to pharynx, consisting of pair of spermiducal bulbs, prostatic vesicle, and penis papilla (Fig. 2A–E). Distal end of each sperm duct forming oval spermiducal bulb, latter having thick muscular wall (Fig. 2A). Distal end of each spermiducal bulb slender and separately connecting to neck of prostatic vesicle (Fig. 2B–D). Prostatic vesicle pear-shaped, having strong muscular wall occupying its proximal one-third, distally coated with connective tissue and enclosed by muscular bulb (Fig. 2F). Canals of extra-vesicular gland penetrating prostatic-vesicle wall. Glandular epithelium with numerous teardrop-shaped cells folded in prostatic vesicle. Ejaculatory duct lacking; distal end of prostatic vesicle directly forming a part of penis papilla. Penis papilla large, conical, and projecting posteriorly into male atrium. Male atrium lined with thin, non-ciliated epithelium.

Pair of oviducts forming common oviduct, which run postero-dorsally to enter vagina (Fig. 2E). From this point, elongated duct of Lang’s vesicle, lined with ciliated epithelium, running posteriorly. Lang’s vesicle sac-shaped, lined with squamous cells, positioned posterior to female gonopore. Vagina lined with smooth ciliated epithelium, running antero-dorsally, curving postero-ventrally as it becomes slenderer, turning postero-dorsally as it becomes wider, eventually leading ventrally to exit at female atrium (or vagina externa). Medial part of vagina surrounded by numerous cement glands (Fig. 2E). Female atrium large, folded, with thick basement membrane opening at female gonopore.

Genital pit with smooth epithelium and basement membrane similar to those in vagina (Fig. 2G), located between male and female gonopores (Fig. 2F, H).

###### Habitat.

Judging from the nature of the dredged material, the sediment type of the species’ habitat is likely to be sandy mud.

###### Molecular phylogeny.

**Figure 3.**
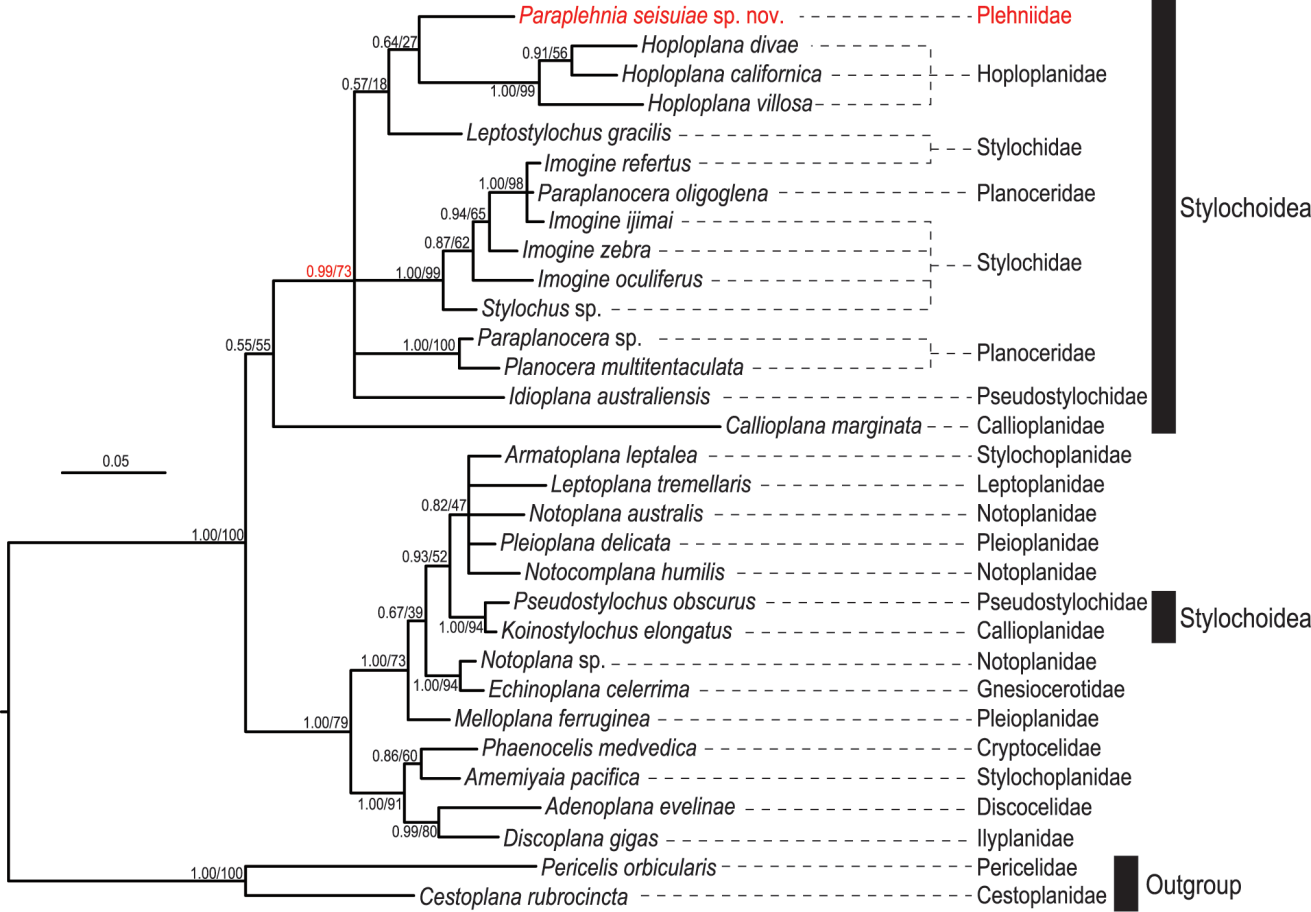
Bayesian phylogenetic tree based on 28S rDNA sequences (603 bp in total). Numbers near nodes are posterior probability and bootstrap value, respectively.

The resulting BI and ML trees were almost identical to each other in topology. *Paraplehniaseisuiae* sp. nov. was nested in a clade composed of stylochoids except *Koinostylochuselongatus* and *Pseudostylochusobscurus* (Fig. 3); the latter two appeared to be more closely related to leptoplanoids than to stylochoids, as indicated by Tsunashima et al. (2017). The majority of stylochoids except *Callioplanamarginata*, *Koinostylochuselongatus*, and *Pseudostylochusobscurus* formed a clade which also included *Stylochus* sp. of Bahia et al. (2017) and was supported by 0.99 BI posterior probability and 73% ML bootstrap (Fig. 3). Given Bahia et al.’s (2017) generic identification of *Stylochus* sp., this clade can be regarded as representing the “true” Stylochoidea, because *Stylochus* is the type genus for this family-group name. While *Paraplehniaseisuiae* sp. nov. appeared as sister to *Hoploplana* spp., its supporting values were low (0.64 BI posterior probability; 27% ML bootstrap). The inter-family relation of Plehniidae among Stylochoidea was thus not fully resolved in the present study.

###### Remarks.

In this paper, we follow the classification system by Prudhoe (1985), in which Plehniidae consists of five genera (*Discocelides*, *Myoramyxa*, *Nephtheaplana*, *Paraplehnia*, and *Plehnia*); for Faubel’s (1983) system, see Discussion below. Hyman (1953) characterized *Paraplehnia* as possessing a prostatic vesicle that has i) a strong muscular wall in its proximal end and ii) a reduced glandular part. *Paraplehniaseisuiae* sp. nov. is characteristic of the genus by possessing these characteristics. By these characters, our new species cannot be placed in *Plehnia* sensu Prudhoe (1985), because the latter is diagnosed to have a prostatic vesicle whose proximal end is not particularly thick-walled. *Paraplehniaseisuiae* sp. nov. differs from *Discocelides* and *Myoramyxa* in that it does not have a vaginal duct (ductus vaginalis) and from *Nephtheaplana* in that our specimen has a pair of spermiducal bulbs.

*Paraplehnia* has contained two species, *P.japonica* (Bock, 1923) and *P.pacifica* (Kato, 1939), and both were originally described from the sublittoral zone in Japan. *Paraplehniaseisuiae* sp. nov. can be distinguished from the two congeners by the thickness of the muscular wall of the prostatic vesicle (about one-third of the prostatic vesicle in *P.seisuiae* sp. nov.; about one-half in *P.japonica* and *P.pacifica*), the presence/absence of a common duct between spermiducal bulbs and prostatic vesicle (absent in *P.seisuiae* sp. nov.; present in *P.japonica* and *P.pacifica*), and the presence/absence of a genital pit between the male and the female gonopores (present in *P.seisuiae* sp. nov.; absent in *P.japonica* and *P.pacifica*) (Table 2). In addition, *P.seisuiae* sp. nov. differs from *P.japonica* by the length of the Lang’s duct (elongated in *P.seisuiae* sp. nov.; short in *P.japonica*) and from *P.pacifica* by the range of developed connective tissues in the female copulatory apparatus (from the female atrium to around the female gonopore and the genital pit in *P.seisuiae* sp. nov.; only around the female atrium in *P.pacifica*).

**Table 2.** Comparison of characters between species of *Paraplehnia*.

	*P.japonica* (Bock, 1923)	*P.pacifica* (Kato, 1939)	*P.seisuiae* sp. nov.
Type locality	Kobe Bay, Japan	Tako-shima, Onagawa, Japan	Sea of Kumano, Japan
Depth	12–15 m	28 m (Kato 1939); 64, 78 m (Hagiya 1993)	298–310 m
Muscular wall in posterior end of prostatic vesicle	About 1/2 of the prostatic vesicle	About 1/2 of the prostatic vesicle	About 1/3 of the prostatic vesicle
Common duct between spermiducal bulbs and prostatic vesicle	Present	Present	Absent
Genital pit	Absent	Absent	Present
Duct of Lang’s vesicle	Short	Elongated	Elongated
Developed connective tissues in the female copulatory apparatus	?	Only around the female atrium	From the female atrium to the genital pit and the female gonopore
Reference	Bock 1923	Kato 1939; Hagiya 1993	This study

The eyespots were invisible in the living specimen (Fig. 1A), probably because of the thickness and opaqueness of the body, as well as the small size of each eyespot. We noticed the presence of eyespots only after sectioning (Fig. 1C, D). Bock (1923: 3) also remarked for *Paraplehniajaponica* that eyespots were undetectable in the living specimens and became apparent only after histological sectioning. Because we failed to observe the arrangement of eyespots from dorsal view in intact body, we had to categorize each eyespot into marginal ones or cerebral ones according to the relative position from the body margin and the brain.

It is for the first time that a genital pit (or genital sucker) was found in a species of plehniid. Among Acotylea, genital pits have been known in *Itanniaornata* Marcus, 1947 (Hoploplanidae Stummer-Traunfels, 1933), three species of *Leptoplana* (Leptoplanidae Stimpson, 1857) (Gammoudi et al. 2012), and *Persicaqeshmensis* Maghsoudlou, Bulnes, & Rahimian, 2015 (Pleioplanidae Faubel, 1983). Genital pits in *I.ornata* are present in a pair, situated on both sides of the female gonopore (Marcus 1952). On the other hand, a single genital pit is present between the male and female gonopores in three *Leptoplana* species and *Persicaqeshmensis*, as well as in *Paraplehniaseisuiae* sp. nov. (Fig. 2H, I).

## Discussion

In this paper, we adopted Prudhoe’s (1985) – instead of Faubel’s (1983) – classification system as to the infrafamilial classification of Plehniidae because this system was followed by some of the subsequent researchers (e.g. Hagiya 1993; Newman and Cannon 1997). Faubel (1983) did not accept *Paraplehnia* because he considered that “the presence (*P.japonica*) or the absence (*P.pacifica*) of Lang’s vesicle demands a separation of both these species” (Faubel 1983: 54–55) and classified *Paraplehniapacifica* as a *Diplehnia*, which was characterized by lacking a Lang’s vesicle (Faubel 1983). However, Kato (1939: 68, fig. 3) clearly stated that the “Lang’s vesicle is small and irregularly elongated, disposed immediately behind the vagina bulbosa in the ventral part of the body” in the original description of *Paraplehniapacifica* and also included a line drawing of the Lang’s vesicle as a schematic figure of the copulatory apparatus of *Paraplehniapacifica*. The validity of *Diplehnia* should be tested by future molecular studies along with *Diplehniacaeca* (Hyman, 1953), the type species of the genus.

Our 28S rDNA analyses corroborate the taxonomic views by Faubel (1983) and Prudhoe (1985) in that Plehniidae, as represented by *Paraplehniaseisuiae* sp. nov. in this study, should be placed in Stylochoidea (Fig. 3), rather than in Cryptoceloidea as Bahia et al. (2017) suggested. Faubel (1983) and Prudhoe (1985) placed Plehniidae in Stylochoidea based primarily on the reproductive-system morphology and the arrangement of eyespots, respectively. Bahia et al. (2017) carried out a 28S-rDNA-based molecular phylogenetic analysis covering 19 families and 32 genera of polyclads. Based on the analysis, Bahia et al. (2017: 674) circumscribed Cryptoceloidea as having “oval to elongated body, without tentacles, and with cerebral, nuchal, and marginal eyespots” and Stylochoidea as having “rounded body, nuchal tentacles, and cerebral, nuchal, and sometimes marginal eyespots”, among other super-familial redefinitions. Bahia et al. (2017: 675) stated that “Polyposthiidae and Plehniidae possibly belong to Cryptoceloidea”, probably because Plehniidae had been defined as having no tentacles (Bock 1913); our new species, *Paraplehniaseisuiae* sp. nov., also lacks tentacles. Litvaitis et al. (2019) inferred the internal relationships of Polycladida using 28S rDNA sequences representing 22 families and 37 genera, and identified morphological characters for each clade recovered. Although Stylochoidea was found to be monophyletic, Litvaitis et al. (2019) concluded that this superfamily cannot be defined by any morphological or developmental synapomorphy. It was because in Litvaitis et al.’s (2019) analyses, Stylochoidea turned out to contain members that lack tentacles (e.g., Latocestidae) and have elongated body (e.g., Latocestidae and *Leptostylochus*), which do not fit to Bahia et al.’s (2017) circumscription for this superfamily. In our analysis, Stylochoidea was “split” into two clades (Fig. 3), and our new species *Paraplehniaseisuiae* sp. nov., having no tentacles, appeared in one of the two stylochoid clades along with *Stylochus* sp. of Bahia et al. (2017). If we suppose that *Paraplehniaseisuiae* sp. nov. is more closely related to *Plehniaarctica* (Plehn, 1896) (originally in *Acelis*; type species of *Plehnia*, which in turn is the type genus for Plehniidae) than any other type species of the type genera of all the nominal families potentially belonging to Stylochoidea, our new species should belong to Plehniidae. If so, and given that Bahia et al.’s (2017) identification of *Stylochus* sp. (see Molecular phylogeny above) was correct, Plehniidae should belong to Stylochoidea. Our study corroborates the opinion of Litvaitis et al. (2019) in that at least the presence/absence of tentacles is not appropriate to circumscribe Stylochoidea.

The inter-familial relation of Plehniidae was not resolved in this study. It is probably due to the shortness of the 28S rDNA sequence (603 bp) employed in the analyses. Future studies should be done with additional molecular makers and more extensive taxon sampling.

## Supplementary Material

XML Treatment for Paraplehnia seisuiae

## References

[B1] Aguado MT Noreña C Alcaraz L Marquina D Brusa F Damborenea C Almon B Bleidorn C Grande C (2017) Phylogeny of Polycladida (Platyhelminthes) based on mtDNA data. Organisms Diversity & Evolution 17(4): 767–778. 10.1007/s13127-017-0344-4

[B2] Akaike H (1974) A new look at the statistical model identification. IEEE Transactions on Automatic Control 19: 716–723. 10.1109/TAC.1974.1100705

[B3] Bahia J Padula V Schrödl M (2017) Polycladida phylogeny and evolution: integrating evidence from 28S rDNA and morphology. Organisms Diversity & Evolution 17(3): 653–678. 10.1007/s13127-017-0327-5

[B4] Bergendal D (1893) Quelques observations sur *Cryptocelidesloveni* mihi. (Note préliminaire). Revue biologique du nord de la France 5: 237–241.

[B5] Bock S (1913) Studien über Polycladen. Zoologiska Bidrag från Uppsala 2: 31–344.

[B6] Bock S (1923) Two new acotylean polyclads from Japan. Arkiv för Zoologi 15(17): 1–41.

[B7] Boom R Sol CJ Salimans MM Jansen CL Wertheim-van Dillen PM van der Noordaa J (1990) Rapid and simple method for purification of nucleic acids. Journal of Clinical Microbiology 28: 495–503. 1691208 10.1128/jcm.28.3.495-503.1990 PMC269651

[B8] Castresana J (2002) Gblocks, v. 0.91b. Online version. http://molevol.cmima.csic.es/castresana/ Gblocks_server.html [Accessed on: 2019-1-18]

[B9] Du Bois-Reymond Marcus E (1965) Drei neue neotropische Turbellaria. Sitzungsberichte der Gesellschaft Naturforschender Freunde zu Berlin (NF) 5: 129–135.

[B10] Faubel A (1983) The Polycladida, Turbellaria. Proposal and establishment of a new system. Part I. The Acotylea. Mitteilungen des hamburgischen zoologischen Museums und Instituts 80: 17–121.

[B11] Felsenstein J (1985) Confidence limits on phylogenies: an approach using the bootstrap. Evolution 39: 783–791. 10.2307/2408678 28561359

[B12] Gammoudi M Egger B Tekaya S Norena C (2012) The genus *Leptoplana* (Leptoplanidae, Polycladida) in the Mediterranean basin. Redescription of the species *Leptoplanamediterranea* (Bock, 1913) comb. nov. Zootaxa 3178(1): 45–56. 10.11646/zootaxa.3178.1.4

[B13] Girard CF (1853) Descriptions of new nemerteans and planarians from the coast of the Carolinas. Proceedings of the Academy of Natural Sciences of Philadelphia 6: 365–367.

[B14] Grube AE (1840) Actinien, Echinodermen und Würmer des adriatischen und Mittelmeers, nach eigenen Sammlungen beschreiben. Verlag von JH Bon, Königsberg, 92 pp 10.5962/bhl.title.23025

[B15] Hagiya M (1993) Note on some polyclad turbellarians (Platyhelminthes) from Otsuchi Bay and its vicinity, Iwate Prefecture. Otsuchi Marine Research Center Report 19: 31–51.

[B16] Haswell WA (1907) Observations on Australian polyclads. Transactions of the Linnean Society of London, Zoology 9(2): 465–485. 10.1111/j.1096-3642.1907.tb00455.x

[B17] Hyman LH (1953) The polyclad flatworms of the Pacific coast of North America. Bulletin of the American Museum of Natural History 100: 269–392.

[B18] Kato K (1934) *Leptostylochusgracilis*, a new polyclad turbellarian. Proceedings of the Imperial Academy 10(6): 374–377. 10.2183/pjab1912.10.374

[B19] Kato K (1937) Polyclads collected in Idu, Japan. Japanese Journal of Zoology 7: 211–232.

[B20] Kato K (1939) Polyclads in Onagawa and vicinity. Science Reports of the Tohoku Imperial University, Fourth Series, Biology 14: 65–79.

[B21] Kato K (1944) Polycladida of Japan. Journal of Sigenkagaku Kenkyusyo 1: 257–319.

[B22] Katoh K Standley DM (2013) MAFFT multiple sequence alignment software version 7: improvements in performance and usability. Molecular Biology and Evolution 30: 772–780. 10.1093/molbev/mst010 23329690 PMC3603318

[B23] Laidlaw FF (1903) On a collection of TurbellariaPolycladida from the Straits of Malacca. (Skeat Expedition 1899–1900). Proceedings of the Zoological Society of London 1: 301–318.

[B24] Lang A (1884) Die Polycladen (Seeplanarien) des Golfes von Neapel und der angrenzenden Meeresabschnitte. Eine Monographie. Engelmann W, Leipzig, 688 pp 10.5962/bhl.title.10545

[B25] Litvaitis MK Bolaños DM Quiroga SY (2019) Systematic congruence in Polycladida (Platyhelminthes, Rhabditophora): are DNA and morphology telling the same story? Zoological Journal of the Linnean Society 20: 1–27. 10.1093/zoolinnean/zlz007

[B26] Maghsoudlou A Bulnes VN Rahimian H (2015) *Persicaqeshmensis* gen. nov. sp. nov from the Persian Gulf (Platyhelminthes: Polycladida: Acotylea), with remarks on reproductive structures. Journal of Natural History 49(25–26): 1477–1491. 10.1080/00222933.2015.1006278

[B27] Marcus E (1947) Turbelários marinhos do Brasil. Boletim da Faculdade de Filosofia, Ciências e Letras da Universidade de São Paulo Zoologia 12: 99–206. 10.11606/issn.2526-4877.bsffclzoologia.1947.125220

[B28] Marcus E (1950) Turbellaria brasileiros (8). Boletim da Faculdade de Filosofia, Ciências e Letras da Universidade de São Paulo Zoologia 15: 69–190. 10.11606/issn.2526-4877.bsffclzoologia.1950.125192

[B29] Marcus E (1952) Turbellaria brasileiros (10). Boletim da Faculdade de Filosofia, Ciências e Letras da Universidade de São Paulo Zoologia 17: 5–186. 10.11606/issn.2526-4877.bsffclzoologia.1952.125189

[B30] Müller OF (1773) Vermium terrestrium et fluviatilium, seu animalium infusoriorum, helminthicorum et testaceorum, non marinorum, succincta historia. Voluminis primi pars prima. Heineck et Faber, Havnia et Lipsia (Copenhagen and Leipzig), 72 pp 10.5962/bhl.title.12733

[B31] Newman LJ Cannon LRG (1997) A new semi-terrestrial acotylean flatworm, *Myoramixapardalota* gen. et sp. nov. (Platyhelminthes, Polycladida) from southeast Queensland. Memoirs of the Queensland Museum 42: 311–314.

[B32] Oya Y Kajihara H (2017) Description of a new *Notocomplana* species (Platyhelminthes: Acotylea), new combination and new records of Polycladida from the northeastern Sea of Japan, with a comparison of two different barcoding markers. Zootaxa 4282(3): 526–542. 10.11646/zootaxa.4282.3.6

[B33] Plehn M (1896) Neue Polycladen, gesammelt von Herrn Kapitan Chierchia bei der Erdumschiffung der Korvett Vettor Pisani, von Herrn Prof. Dr. Kukenthal im nördlichem Eismeer und von Herrn Prof Dr. Semon in Java. Jenaische Zeitschrift für Naturwissenschaft 30: 137–181.

[B34] Poche F (1926) Das System der Platodaria. Archiv für Naturgeschichte Abteilung A 91: 1–458.

[B35] Prudhoe S (1985) A Monograph on Polyclad Turbellaria. Oxford University Press, Oxford, 253 pp.

[B36] Ronquist F Huelsenbeck JP (2003) MrBayes 3: Bayesian phylogenetic inference under mixed models. Bioinformatics 19: 1572–1574. 10.1093/bioinformatics/btg180 12912839

[B37] Schmarda LK (1859) Neue wirbellose Thiere beobachtet und gesammelt auf einer Reise um die Erde 1853 bis 1857. Bd. I: Turbellarien, Rotatorien und Anneliden. Engelmann W, Leipzig, 66 pp 10.5962/bhl.title.85313

[B38] Sonnenberg R Nolte AW Tautz D (2007) An evaluation of LSU rDNA D1–D2 sequences for their use in species identification. Frontiers in Zoology 4(1): 1–12. 10.1186/1742-9994-4-6 17306026 PMC1805435

[B39] Stamatakis A (2014) RAxML version 8: a tool for phylogenetic analysis and post-analysis of large phylogenies. Bioinformatics 30: 1312–1313. 10.1093/bioinformatics/btu033 24451623 PMC3998144

[B40] Stimpson W (1857) Prodromus descriptionis animalium evertebratorum, quae in Expeditione ad Oceanum Pacificum Septentrionalem a Republica Federata missa, Johanne Rodgers Duce, observavit et descripsit. Pars I, Turbellaria Dendrocoela. Proceedings of the Academy of Natural Sciences of Philadelphia 9: 19–31. 10.5962/bhl.title.51447

[B41] Stummer-Traunfels R (1933) Polycladida. In: Bronn HG (Ed.) Klassen und Ordnungen des Tierreichs, Vierter Band, Abteilung 1c (Volume 4, Part 1c).Akademische Verlagsgesellschaft, Leipzig, 3485–3596.

[B42] Tamura K Peterson D Peterson N Stecher G Nei M Kumar S (2011) MEGA5: molecular evolutionary genetics analysis using maximum likelihood, evolutionary distance, and maximum parsimony methods. Molecular Biology and Evolution 28: 2731–2739. 10.1093/molbev/msr121 21546353 PMC3203626

[B43] Tanabe AS (2011) Kakusan4 and Aminosan: two programs for comparing nonpartitioned, proportional and separate models for combined molecular phylogenetic analyses of multilocus sequence data. Molecular Ecology Resources 11(5): 914–921. 10.1111/j.1755-0998.2011.03021.x 21592310

[B44] Tsunashima T Hagiya M Yamada R Koito T Tsuyuki N Izawa S Kosoba K Itoi S Sugita H (2017) A molecular framework for the taxonomy and systematics of Japanese marine turbellarian flatworms (Platyhelminthes, Polycladida). Aquatic Biology 26: 159–167. 10.3354/ab00682

[B45] Verrill AE (1882) Notice of the remarkable marine fauna occupying the outer banks off the southern coast of New England, no. 7, and of some additions to the fauna of Vineyard Sound. American Journal of Science 24: 1–360. 10.2475/ajs.s3-24.143.360

[B46] Woodworth WM (1898) Some planarians from Great Barrier Reef of Australia. Bulletin of the Museum of Comparative Zoology of Harvard College 32(4): 61–67.

[B47] Yeri M Kaburaki T (1918) Bestimmungsschlüssel für die japanischen Polycladen. Annotationes Zoologicae Japonenses 9: 431–442.

